# Emergency critical care: closing the gap between onset of critical illness and intensive care unit admission

**DOI:** 10.1007/s00508-024-02374-w

**Published:** 2024-05-16

**Authors:** Martin W. Dünser, Matthias Noitz, Thomas Tschoellitsch, Markus Bruckner, Markus Brunner, Bernhard Eichler, Romana Erblich, Stephan Kalb, Marius Knöll, Johannes Szasz, Wilhelm Behringer, Jens Meier

**Affiliations:** 1 Department of Anesthesiology and Intensive Care Medicine, Kepler University Hospital and Johannes Kepler University, 4020 Linz, Austria; 2 Ambulance and Disaster Relief Services, Oberösterreichisches Rotes Kreuz, 4020 Linz, Austria; 3 Department of Emergency Medicine, Vienna General Hospital, 1090 Vienna, Austria; 4 Department of Anesthesiology and Intensive Care Medicine, Kepler University Hospital and Johannes Kepler University, Krankenhausstraße 9, 4020 Linz, Austria

**Keywords:** Emergency critical care, Critical illness, Prehospital, Emergency department, Medical emergency team

## Abstract

Critical illness is an exquisitely time-sensitive condition and follows a disease continuum, which always starts before admission to the intensive care unit (ICU), in the majority of cases even before hospital admission. Reflecting the common practice in many healthcare systems that critical care is mainly provided in the confined areas of an ICU, any delay in ICU admission of critically ill patients is associated with increased morbidity and mortality. However, if appropriate critical care interventions are provided before ICU admission, this association is not observed. Emergency critical care refers to critical care provided outside of the ICU. It encompasses the delivery of critical care interventions to and monitoring of patients at the place and time closest to the onset of critical illness as well as during transfer to the ICU. Thus, emergency critical care covers the most time-sensitive phase of critical illness and constitutes one missing link in the chain of survival of the critically ill patient. Emergency critical care is delivered whenever and wherever critical illness occurs such as in the pre-hospital setting, before and during inter-hospital transfers of critically ill patients, in the emergency department, in the operating theatres, and on hospital wards. By closing the management gap between onset of critical illness and ICU admission, emergency critical care improves patient safety and can avoid early deaths, reverse mild-to-moderate critical illness, avoid ICU admission, attenuate the severity of organ dysfunction, shorten ICU length of stay, and reduce short- and long-term mortality of critically ill patients. Future research is needed to identify effective models to implement emergency critical care systems in different healthcare systems.

## The continuum and time sensitivity of critical illness

Critical illness is a potentially reversible state of ill health with unstable vital functions and/or vital organ dysfunction, which carries a high risk of imminent death if care is not provided [1]. Critical illness affects all age groups and can be the consequence of a multitude of acute and chronic conditions. It follows a disease continuum, which always starts before admission to the intensive care unit (ICU), in the majority of cases even before hospital admission [2]. While some critical conditions occur within minutes or even seconds, others develop more gradually either complicating acute diseases or resulting from decompensation of chronic illness (Table 1). Independent of its onset, all types of critical illness share one characteristic feature: the need for timely critical care interventions. Cardiac arrest [3], airway obstruction [4], brain impact apnoea [5], acute haemorrhage [6], myocardial infarction [7], stroke [8], secondary peritonitis [9], or sepsis [10] are just a few conditions underlining the exquisite time sensitivity of critical illness.

**Table 1:** Types of critical illness: Typical settings in which critical illness occurs, associated main challenge and examples

	Acute Critical Illness	Critical Illness Complicating Acute Disease	Critical Illness Complicating Chronic Disease
Typical Setting	Pre-hospital	Pre-hospital, ED, ward	Pre-hospital, ED, ward
Main Challenge	Timely delivery of critical care	Timely recognition of critical illness	Timely recognition of critical illness
Examples	Major trauma	Sepsis	COPD exacerbation
	Cardiac arrest	Acute respiratory distress syndrome	Acute-on-chronic heart failure
	Gastrointestinal haemorrhage	Acute kidney injury	Acute-on-chronic liver failure
	Acute heart failure (incl. acute coronary syndrome and arrhythmia)	Delirium	Acute-on-chronic kidney failure
	Stroke	Complications of acute surgical disease	Paraneoplastic complications
	Seizure/status epilepticus	Metabolic derangements (e.g., ketoacidosis, hyperglycaemia, electrolyte disturbances)	Drug-induced organ toxicity
	Anaphylaxis		Rejection following organ transplantation
	Poisoning		

## The gap between onset of critical illness and ICU admission

Critical care is widely considered as the care provided in specially equipped and staffed geographic areas of a hospital, namely ICUs. Although newer definitions of critical care and ICUs acknowledge that the activities of the critical care team often extend beyond the walls of the physical space of the ICU [11], it is still common practice in many healthcare systems to confine critical care delivery to ICUs. This practice, however, stands in striking contrast to the time sensitivity of critical illness.

Supporting the abovementioned assumption that critical care is mainly provided in ICUs, there is culminating evidence indicating that delays in admitting critically ill patients to the ICU are associated with increased morbidity and mortality. In a single centre study from the United States, delayed ICU transfer of critically ill ward patients resulted in a higher degree of organ dysfunction at ICU admission and an almost 4fold increase in hospital mortality [12]. Similarly, longer boarding times of critically ill patients in the emergency department were associated with persistent organ dysfunction, more resource use, prolonged ICU length of stay and higher odds of in-hospital mortality [13–22]. In a general hospital population, each one-hour increase in the delay to ICU admission resulted in a 3% rise in the adjusted odds for death of critically ill patients [23]. A systematic review and meta-analysis including 34 studies with a low risk of bias concluded that delayed ICU admission was independently associated with higher mortality rates. The pooled odds ratio for mortality in case of delayed ICU admission was 1.61 (CI95%, 1.44–1.81). In a subgroup analysis, the risk of death due to delayed ICU admission was remarkably higher in postoperative compared to non-surgical patients (OR 2.44; CI95%, 1.49–4.01) [24].

The observed association between delays in ICU transfer and adverse outcomes of critically ill patients likely has two main reasons: 1) limited ICU capacities resulting in prolonged delays in ICU admission of critically ill patients [19, 25–28], and 2) provision of inadequate care to critically ill patients before ICU admission. Both factors expose a delicate management gap of the critically ill patient between the onset of critical illness and ICU admission. Given economic restrictions [29] and staff shortages [30], it is unlikely that ICU capacities can be increased to such an extent that critically ill patients can be admitted without delay in all settings and at all times. Even if ICUs hypothetically had unlimited capacities the need to deliver critical care to patients from the onset of critical illness to ICU admission would remain a challenge. The current literature suggests that adequate delivery of critical care before ICU admission is hampered by both delayed recognition of critical illness [31–35] and under-resuscitation. A retrospective cohort study from Canada for example reported that although critically ill patients were managed in emergency departments for significant lengths of time, relatively few of them underwent invasive procedures while in the emergency department [36].

## The concept of emergency critical care

**Fig. 1:**
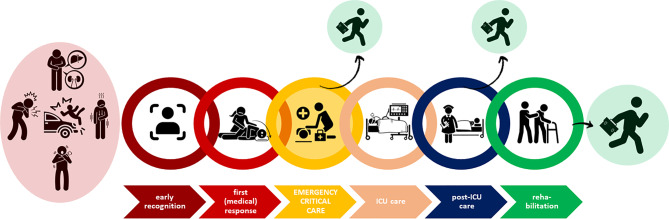
The chain of survival of critically ill patients illustrating the link of emergency critical care as well as pathways to recovery from critical illness. (*ICU* intensive care unit). (Images by flaticon.com)

**Fig. 2:**
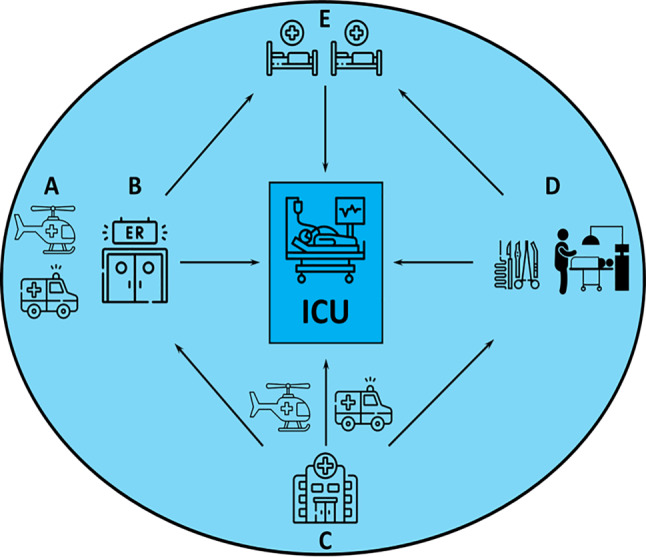
Spectrum of the settings in which emergency critical care interventions are delivered to critically ill patients outside of the ICU. (**a** pre-hospital critical care, **b** critical care in the emergency department, **c** retrieval and interhospital transfer of critically ill patients, **d** intraoperative critical care, **e** critical care provided by medical emergency or rapid response teams on hospital wards; *ER* emergency room, *ICU* intensive care unit). (Images by flaticon.com)

**Fig. 3:**
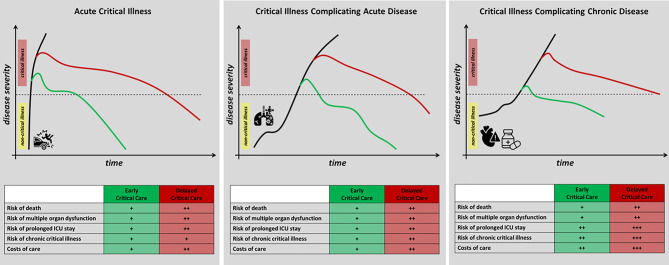
Schematic presentation of the potential effects of early vs. delayed critical care interventions in patients with acute critical illness, critical illness complicating acute disease and critical illness complicating chronic disease. (Black lines represent the natural disease course, green lines represent the disease course when critical care interventions are delivered early (e.g., at the closest time point and location of the onset of critical illness), and red lines represent the disease course when critical care interventions are delayed (e.g., initiated only after intensive care unit admission). The figure schematically summarizes scientific evidence summarized in the section “Scientific Evidence”; *ICU* intensive care unit)

Emergency critical care refers to critical care provided outside of the ICU. It encompasses the delivery of critical care interventions to and monitoring of patients at the place and time closest to the onset of critical illness as well as during transfer to the ICU. Thus, emergency critical care covers the most time sensitive phase of critical illness and constitutes one missing link in the chain of survival of the critically ill patient (Fig. 1). Emergency critical care is delivered whenever and wherever critical illness occurs such as in the pre-hospital setting (e.g., at the site of the accident or where acute critical illness occurs), before and during inter-hospital transfers of critically ill patients, in the emergency department, in the operating theatres, and on hospital wards (Fig. 2). By closing the management gap between onset of critical illness and ICU admission, emergency critical care can not only prevent early deaths, attenuate organ dysfunction and enhance early recovery, but also reverse mild and moderate forms of critical illness (Fig. 3).

The principles of emergency critical care closely resemble those of critical care provided in the ICU. However, certain aspects are more relevant during the acute phases of critical illness than during the stabilization and recovery phases generally encountered on the ICU. Aside from early resuscitation and interventions to stabilize the airway, breathing and circulation, one crucial aspect of emergency critical care is the diagnostic work-up of the underlying condition leading to critical illness. Only when the causative pathology has been identified correctly and treated adequately, can critical illness be efficiently reversed [37]. Although the diagnostic work-up of critically ill patients uses similar techniques as in non-critically ill patients, two important differences exist: First, compared to the non-critically ill patient, the sequence of history taking, physical examination, and diagnostic tests requires adjustment to the need for immediate resuscitation. In addition, many critically ill patients are not capable of giving an extensive history of their present illness. Therefore, history taking commonly relies on relatives, carers, or bystanders. It is crucial to highlight that the basic process to arrive at the diagnosis does not differ between critically ill and non-critically ill patients. Importantly, the two fundamental diagnostic tools of medicine (history taking and careful physical examination) must not be replaced by laboratory screening and over-aggressive use of radiological imaging too often rendering false positive results. Second, in line with the time sensitivity of critical illness the diagnostic work-up needs to be conducted without delays and in concert with resuscitation efforts. Common methods to minimize the time for the diagnostic work-up of critically ill patients include bedside ultrasound, point-of-care laboratory testing, mobile x‑rays, as well as minimizing waiting times until imaging techniques such as computer tomography or rarely magnetic resonance imaging [38].

## Historical perspective

The concept of emergency critical care is not new. Fifty years ago, Peter Safar, father of cardiopulmonary reanimation and resuscitation pioneer, referred to critical care medicine as the triad of resuscitation, emergency care of life-threatening conditions, and ICU therapy. Safar highlighted that critical care needs to overcome the borders of ICUs and embrace all components of the system which the critically ill or injured patient needs including pre-hospital care, transportation with life support, as well as critical care delivered in the emergency department and operating room. Without adequate critical care delivered before ICU admission, Safar stated ICU care often becomes unnecessarily expensive terminal care [39]. In 1991, the Emergency Department at the Vienna General Hospital opened and was one of the first facilities to incorporate an ICU within the emergency department. This setting was referred to serve as the closing link in the chain of survival for critically ill patients [40]. Approximately 20 years ago, the ‘ICU without walls’ or ‘ICU without borders’ concept was founded in Australia [41]. This concept focused on the early recognition and adequate care of patients developing critical illness on hospital wards [42]. It led to the implementation of medical emergency or rapid response systems. The authors feel that the term emergency critical care is more inclusive and better covers the care required during the entire continuum of critical illness before ICU admission encompassing the concepts suggested by Peter Safar as well as the ‘ICU without walls’ model.

## The global perspective on emergency critical care

In May 2023, the 76th World Health Assembly passed a resolution calling for timely efforts to strengthen the planning and provision of emergency, critical care and operative services, a system that represents the first-tier of care for critically ill patients [43]. This resolution aims to ensure high-quality holistic critical care around the globe by removing barriers to the expansion of emergency and critical care systems. A group of health ministers and international stakeholders underlined that emergency, critical and operative care represents a people-centred continuum, and that the delivery of these services is not associated with a single medical profession, specialty, condition or setting. On a global scale, nurses, technicians, and paramedics deliver as much emergency and critical care as doctors, and all may be trained as specialists of the first hours of care [44]. Using a Delphi process, 269 international experts concluded that a package to deliver emergency and essential critical care contains 40 clinical processes (e.g., identification and care of critical illness as well as general processes of care) and 67 requirements (e.g., equipment, consumables, drugs, human resources, training, routines, guidelines, infrastructure) [45]. Another global group of anaesthetists and critical care physicians underlined that in most areas of the world it is unrealistic that state-of-the-art ICU care can be provided to all critically ill patients leaving emergency critical care as the most widely available, cost-effective and resource-sparing form of critical care delivered around the world [46].

## Training and education

The World Federation of Societies of Intensive and Critical Care Medicine refers to critical care as a multidisciplinary and interprofessional specialty dedicated to the comprehensive care of critically ill patients [11]. In line with this definition, it is important to understand that emergency critical care is a concept and not part of one medical specialty. Neither is it bound to one medical profession, as in many countries pre-hospital critical care is routinely delivered by paramedics or specially trained nurses. Depending on the individual setting, emergency critical care may be delivered by the staff from the ICU, emergency department, anaesthesiology department, surgery department, or other relevant departments. Independent of which team provides emergency critical care, the common denominator among all providers including doctors and nurses must be adequate training and experience to care for critically ill patients. Several educational bodies have responded to this need and provide dual specialist training in emergency and critical care medicine [47].

## Scientific evidence

A scoping review of the literature (via Medline [Ovid]) without language, age or publication date restrictions was conducted in order to identify models to deliver critical care outside of the ICU as well as scientific evidence on relevant and patient-centred outcome effects of emergency critical care interventions. We deliberately did not search for published evidence on critical care delivery during the intraoperative phase as this is an established concept [48, 49] and an integral part of the medical specialty of anaesthesia.

### Prehospital critical care

The outcome effects of critical care interventions provided in the pre-hospital setting have mainly been evaluated in major trauma victims and patients suffering from sudden unexpected cardiac arrest. Severe trauma patients who underwent pre-hospital critical care interventions, including endotracheal intubation, chest decompression, tourniquet use, cricothyroidotomy, and advanced cardiac life support, had a lower mortality than those who did not [50]. The Head Injury Retrieval Trial suggested that critical care interventions delivered in the pre-hospital setting reduced 30-day mortality by 30% (number needed to treat, 6) in adult patients with severe blunt head injury when compared with standard ground paramedic management [51]. In a retrospective cohort study conducted in the United Kingdom, patients who experienced a sustained return of spontaneous circulation following traumatic cardiac arrest received more critical care interventions on scene than trauma victims without a return of spontaneous circulation. Delivery of bag-valve-mask ventilation, rapid sequence induction, blood product administration, and thoracostomies were independently associated with a sustained return of spontaneous circulation [52]. In a French multicentre study including 2703 blunt trauma patients, prehospital interventions delivered by a mobile critical care team (e.g., venous line, crystalloid or colloid infusions, mannitol, catecholamines, tracheal intubation, mechanical ventilation, blood products, chest tube) reduced 30-day mortality [53]. Although controversial data exist [54], prehospital intubation was associated with a lower risk of death and better functional outcome at six months compared to no prehospital intubation in patients with severe traumatic brain injury [55, 56]. The authors of a recent meta-analysis of 19 studies concluded that there is growing evidence that pre-hospital endotracheal intubation in patients with severe traumatic brain injury was beneficial if performed by well-trained, experienced providers in accordance with current guidelines [57].

In a prospective, observational multicentric study conducted in the United Kingdom, prehospital critical care interventions resulted in a higher rate of hospital admission in patients with out-of-hospital cardiac arrest compared to routine advanced life support. However, this did not translate into increased rates of survival to hospital discharge [58]. Delayed arrival of teams able to provide advanced life support at the scene adversely affected neurological outcomes at hospital discharge in adults experiencing an out-of-hospital cardiac arrest [59]. In the pre-hospital setting, even complex procedures such as veno-arterial ECMO therapy can be delivered to selected patients with out-of-hospital cardiac arrest [60]. Following first pre-hospital eCPR systems in Paris [61] and Regensburg/Germany [62], programs systematically implementing pre-hospital eCPR have reported good results with favourable neurological survival rates in 43% of patients at hospital discharge and three months thereafter [63].

### Critical care retrieval and interhospital transfer of critically ill patients

Critical care retrieval or retrieval medicine are the terms used to describe the use of an expert team to assess, stabilise and transport critically injured or ill patients from one medical facility to a higher level of care [64]. The term ‘retrieval medicine’ originates from the continent of Australia, where the practice of transferring critically ill patients over long distances is common and highly developed [65]. In other regions of the world, retrieval medicine is also referred to as interhospital transfer of critically ill patients.

Several observational studies indicated that interhospital transports of critically ill patients can safely be accomplished when performed by specialized and adequately staffed critical care transport teams. In a retrospective review of inter-hospital transfers in the United Kingdom, the use of a specialist transfer team improved acute physiology of critically ill patients compared with standard ambulance transport accompanied by a doctor provided by the referring hospital [66]. Similarly, inter-hospital transfers of critically ill patients using a specialized mobile ICU transfer team resulted in less adverse technical events, less deterioration of pulmonary function [67] and a lower 24-hour mortality [68] than standard ambulance transfers. Two studies revealed that the mortality of critically ill patients requiring inter-hospital transfer was not different from non-transferred patients when the inter-facility transport was conducted by a dedicated retrieval team adhering to ICU relocation protocols [69, 70]. Specialized retrieval teams can implant extracorporeal life support systems in the referring hospital before inter-hospital transfer in patients with severe respiratory and/or cardiovascular failure who may otherwise be too unstable for transportation [71–73], a practice likely associated with mortality benefits [74]. The ability of specially trained critical care teams to safely transport critically injured or ill patients even over long distances (e.g., during intercontinental flights) has been shown both in civilian and military settings [75–77].

Use of specially trained paediatric critical care transport teams is of particular benefit for neonatal and paediatric inter-hospital transport [78–80]. Such teams are considered a prerequisite for the policy of centralizing paediatric intensive care services [79]. In this context, an analysis of prospectively collected data in England indicated that paediatric ICU retrieval services often needed to stabilize critically ill children before inter-hospital transport and that the additional time required to do so was not associated with worse outcome [81].

### Critical care in the emergency department

The emergency department is the first point of contact for acutely ill patients presenting to the hospital. Aside from the ICU and operating theatres, it is also the hospital area, where most critically ill patients are encountered. Several models to provide critical care in the emergency department and improve the management of critically ill emergency patients have been published [82]. Such critical care delivery solutions substantially vary from each other and include medical emergency [83] or emergency critical care teams [84] bringing ICU staff to the ED whenever needed, ED-based early intervention teams [85, 86], ICU-led telemonitoring solutions [87], dedicated critical care resuscitation units [88, 89], and emergency physician-staffed emergency department ICUs [40, 90–94].

Multiple studies reported that critical care delivered to critically ill patients in the emergency department (e.g., short-term non-invasive pressure support ventilation in patients with cardiogenic pulmonary oedema) can rapidly stabilize organ functions, halt or reverse progression of organ dysfunction, and avoid the need for ICU admission in emergency department patients who initially presented with critical illness [95–97]. Importantly, the time duration critically ill patients stayed in the emergency department before ICU admission was not negatively associated with patient outcomes when appropriate critical care was delivered in the emergency department [98, 99]. The most important evidence-based benefits of critical care delivery in the emergency department are summarized in Table 2 [100]. In patients too old, frail, and/or sick to benefit from any critical care intervention, critical care delivery in the emergency department includes provision of excellent and humane palliative care [101, 102]. Identification of patient preferences in both critically and non-critically ill patients before ICU and hospital admission is another patient-centred and important aspect of critical care delivery in the emergency department. An economic analysis revealed that critical care delivery in the emergency department is cost-effective [103].

**Table 2:** Overview of evidence-based benefits of providing critical care in the emergency department.#

Increased rate of timely critical care interventions delivered to critically ill patients in the ED [83, 90, 91]
Reduced rate of ICU admissions for critically ill ED patients, particularly for patients with mild to moderate disease severity including patients with a need for continuous monitoring, gastrointestinal haemorrhage, or sepsis [84, 87, 90, 93, 97, 98, 130–132]
Reduced rate of ICU admissions for patients transferred from the ED to non-ICU wards [90, 132]
Reduced ICU length of stay for ED patients requiring ICU admission [90, 132, 133]
Increased ICU capacities for non-ED patients [131]
Reduced length of stay in the hospital [130, 132]
Reduction of mortality at 24 h, ICU discharge, hospital discharge, 30 days, 60 days, and 365 days for critically ill ED patients [84, 87, 90, 131, 132]

*ED* emergency department, *ICU* intensive care unit

### Critical care provided by medical emergency or rapid response teams on hospital wards

**Fig. 4:**
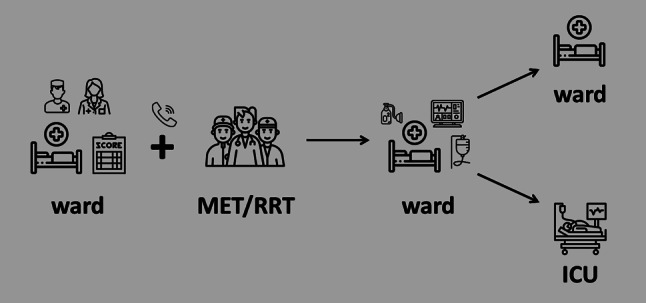
Schematic description of a medical emergency or rapid response system consisting of an afferent (e.g., ward team recognizing critical illness using clinical acumen and validated scores) and efferent loop (e.g., medical emergency or rapid response team providing critical care support). (*ICU* intensive care unit, *MET* medical emergency team, *RRT* rapid response system). (Images by flaticon.com)

Clinical deterioration and cardiac arrest in patients on general wards are frequently preceded by physiologic derangements which can be identified with the use of scores such as the National Early Warning Score [104–108]. These observations led to the implementation of medical emergency or rapid response systems (Fig. 4). Such teams typically consist of critical care physicians, critical care nurses, physiotherapists and/or respiratory therapists. A systematic review found no relationship between team composition and patient outcomes, but highlighted that mature and dedicated teams which required mandatory activation reported the best results [109]. Hypoxaemia (41%), arterial hypotension (28%), an altered conscious state (23%), tachycardia (19%), tachypnoea (14%), and oliguria (8%) were the most frequent alarm triggers leading to activation of the medical emergency team in an Australian hospital [110]. In a study from Portugal, fluid challenges (40.6%), bag mask ventilation (37.3%), intravenous access (29.8%), manual ventilation (20.1%), endotracheal intubation (15.7%), airway suction (13.6%), and cardiopulmonary resuscitation (12.3%) were the most common critical care interventions delivered by the medical emergency team on the ward. The median on-scene time of the team was 35 (IQR, 20–50) minutes [111]. An Italian study reported that critical care (e.g., helmet continuous positive airway pressure, pharmacological cardiovascular support) provided to critically ill haematological patients on wards allowed for stabilization and avoidance of ICU admission in one third of patients [112]. A retrospective chart review conducted in a US hospital revealed that implementation of a rapid response system was associated with an increase in do-not-resuscitate order placements. Furthermore, medical emergency team activation fostered discussions on goals of care and frequently resulted in transition to a palliative care strategy [113].

A retrospective study from a Swiss tertiary centre found that an increase in the number of medical emergency team calls from 5.2 to 16.5 per 1000 hospital admissions paralleled a decrease in cardiac arrest calls from 1.6 to 0.8 per 1000 admissions [114]. Similarly, a post hoc analysis of a cluster randomized controlled trial reported that an increasing proportion of emergency team calls was inversely related to the rate of cardiac arrests and unexpected deaths [115]. Introduction of a rapid response team decreased unexpected fatalities translating in an estimated 1.5 lives saved per week in a French hospital. Overall, in-hospital mortality decreased from 39.6 to 34.6 per 1000 hospital discharges [116]. Several authors reported on reductions in the rates of unexpected cardiac arrests, unexpected ICU admissions and re-admissions, as well as both short- and long-term mortality following medical emergency or rapid response team implementation [111, 117–122]. The nationwide implementation of rapid response systems in the Netherlands was associated with a decrease in cardiac arrests, unplanned ICU admissions, and in-hospital mortality in adult ward patients [123]. In line with these results, three meta-analyses reported that implementation of medical emergency or rapid response systems reduced the rates of cardiac arrests outside of the ICU and in-hospital mortality [124–126]. Late activations of rapid response teams were associated with increased rates of ICU admission, prolonged hospital stays and higher mortality [127]. The European Resuscitation Council Guidelines 2021 recommended rapid response systems as the efferent loop in the modified ‘in-hospital chain of survival’ to prevent in-hospital cardiac arrest [128].

## Research opportunities

The concept of emergency critical care opens a wide range of research opportunities. In line with the clinical spectrum of emergency critical care paralleling the continuum of critical illness, emergency critical care expands critical care research from the ICU to earlier phases of critical illness. Delayed initiation of study interventions (e.g., only after ICU admission) may be one reason why some putatively effective therapies had not translated into improved patient outcomes when evaluated in randomized controlled trials [129]. Further research is required to identify effective strategies to deliver emergency critical care. In view of widespread organizational heterogeneity in the care of critically ill patients outside of ICUs, it is highly likely that there is no one-size-fits-all template but rather different models to implement the concept of emergency critical care in different healthcare systems.

## Conclusions and outlook

Emergency critical care refers to the concept of delivering critical care interventions outside of the ICU at the closest time and location to the onset of critical illness. By providing critical care interventions to critically ill patients in the pre-hospital setting, during inter-hospital transfers, in the emergency department, operating theatres, or on hospital wards, emergency critical care closes the current management gap between the onset of critical illness and ICU admission. Scientific evidence suggests that emergency critical care interventions improve patient safety and can avoid early deaths, reverse mild-to-moderate critical illness, avoid ICU admission, attenuate the severity of organ dysfunction, shorten ICU length of stay, and reduce short- and long-term mortality of critically ill patients. Future research is needed to identify effective models to implement emergency critical care systems in different healthcare systems.
